# Policy choices for Shanghai responding to challenges of Omicron

**DOI:** 10.3389/fpubh.2022.927387

**Published:** 2022-08-09

**Authors:** Ying Qian, Siqi Cao, Laijun Zhao, Yuge Yan, Jiaoling Huang

**Affiliations:** ^1^Business School, University of Shanghai for Science and Technology, Shanghai, China; ^2^School of Public Health, Shanghai Jiao Tong University School of Medicine, Shanghai, China

**Keywords:** COVID-19, Omicron, system dynamics modeling, intervention policies, simulation

## Abstract

**Background:**

A new wave of Coronavirus disease 2019 (COVID-19) infection driven by Omicron BA.2 subvariant hit Shanghai end of February 2020. With higher transmissibility and milder symptoms, the daily new confirmed cases have soared to more than 20 K within one and a half months. The greatest challenge of Omicron spreading is that the rapidly surging number of infected populations overwhelming the healthcare system. What policy is effective for huge cities to fight against fast-spreading COVID-19 new variant remains a question.

**Methods:**

A system dynamics model of the Shanghai Omicron epidemic was developed as an extension of the traditional susceptible-exposed-infected-susceptible recovered (SEIR) model to incorporate the policies, such as contact tracing and quarantine, COVID-19 testing, isolation of areas concerned, and vaccination. Epidemic data from Shanghai Municipal Health Commission were collected for model validation.

**Results:**

Three policies were tested with the model: COVID-19 testing, isolation of areas concerned, and vaccination. Maintaining a high level of COVID-19 testing and transfer rate of the infected population can prevent the number of daily new confirmed cases from recurring growth. In the scenario that 50% of the infected population could be transferred for quarantine on daily bases, the daily confirmed asymptomatic cases and symptomatic cases remained at a low level under 100. For isolation of areas concerned, in the scenario with most isolation scope, the peak of daily confirmed asymptomatic and symptomatic cases dropped 18 and 16%, respectively, compared with that in the scenario with least isolation. Regarding vaccination, increasing the vaccination rate from 75 to 95% only slightly reduced the peak of the confirmed cases, but it can reduce the severe cases and death by 170%.

**Conclusions:**

The effective policies for Omicron include high level of testing capacity with a combination of RAT and PCR testing to identify and quarantine the infected cases, especially the asymptomatic cases. Immediate home-isolation and fast transfer to centralized quarantine location could help control the spread of the virus. Moreover, to promote the vaccination in vulnerable population could significantly reduce the severe cases and death. These policies could be applicable to all metropolises with huge population facing high transmissible low severity epidemic.

## Introduction

A new wave of Coronavirus disease 2019 (COVID-19) infection driven by the new variant, Omicron, started in January 2020. Global countries are now facing new challenges brought by Omicron. Current studies consistently showed that this new variant is substantially more transmissible than the Delta variant, capable of significant immune evasion, and with milder or even without symptoms (1–3). The effectiveness of vaccination against infection dropped dramatically suggested by current studies (3, 4), even to 33% reported by one study (5). WHO reported this February that 130 million cases and 500,000 deaths had been recorded globally since Omicron was declared a variant of concern in late November, calling the count “beyond tragic.” Latest data showed that the WHO's Europe region accounted for 58% of new confirmed cases, and 35% of new deaths, and the Americas made up 23% of new cases and 44% of new deaths (6). The huge challenge with the steep rise of infections of Omicron is the overwhelmed and understaffed healthcare system. Specialists warned that a considerable overload of the hospitals is to be expected (7). Based on a mathematical model of SARS-CoV-2 transmission tailored to the unique immunization and epidemiological situation of China, Yu concluded that the level of immunity induced by the current vaccination campaign would be insufficient to prevent overwhelming the healthcare system and major losses of human lives (8). Take the UK as an example, in England, more than 650,000 people have probably been infected twice; most of them were re-infected in the past 2 months, according to data collected by the UK Health Security Agency (9, 10). The British Medical Association reported that the facts, figures, and the living reality for thousands of patients and the National Health Service (NHS) staff daily demonstrate undoubtedly that the NHS is currently already overwhelmed (11). Alderwick pointed out that millions of people are already feeling the unbearable strain in the UK health system (12).

Before Omicron, strict intervention protection controls (IPCs) were adopted by China after the outbreak of COVID-19, such as active case surveillance at fever clinics, immediate isolation of cases, quarantine of close contacts and high-risk groups, polymerase chain reaction (PCR) testing, and compulsory use of masks in the general population (13). China's strict policy preference, called zero-COVID strategy, was corroborated by WHO of the outbreak dynamic and case count reported by the Chinese government (14), demonstrating that a strict and rapid response to an emerging epidemic can halt the spread of a new virus (15). The previous study reported that the zero-COVID strategy was estimated to have saved one million lives, compared with the global average mortality of COVID-19 (as of 16 February 2022) (16). This zero-COVID policy is undoubtedly successful containing the pandemic prior to the current outbreak caused by Omicron. However, this policy is being challenged as a new wave of Omicron hits. Recently, China's National Health Commission changed its rules so that mild cases could be isolated in centralized locations, rather than treated in hospitals, and the criteria for a patient to be discharged from isolation have also been lowered, which has aroused extensive attention and discussion. There have been arguments that it is time to prepare for relaxing the policy. Chen and Chen believed that this change will happen sooner or later, as SARS-CoV-2 will probably become a seasonal infection in 2022 and circulate in humans indefinitely (17).

In Shanghai, the timely and precise strategies taken to prevent the spread of COVID-19 had been successful in the past 2 years (18). Active monitoring, precise and fast contact tracing, and timely PCR testing for related population helped interrupt the transmission of virus with minimal social economic impact. However, in this wave of Omicron BA.2 subvariant infection, which started from February 24^th^ 2022 with one asymptomatic case, the number of daily new confirmed cases soared to more than 20 K within one and a half months. From March 12^th^, all primary and secondary schools stopped offline classes and switched to online learning. On March 27^th^, extending the IPC policy of isolating high-risk areas, the Shanghai government announced a two-stage home isolation of the whole city, while PRC testing was carried out city-wide. About 9 million residents of Pudong, the eastern half of Shanghai, have been home isolated since March 28^th^. On the other side of the bund, roughly 15 million people in the west of the city, initiated a home isolation policy since April 1^st^ 2021. After almost 2-week home isolation, on April 9^th^, the authority announced that the city's residential compounds. Villages and business locations will be classified into three types of zones: isolation, control, and precaution. Shanghai, as a metropolis with huge population, faced extreme challenges in this wave of Omicron BA.2 subvariant. A series of questions remained unanswered, for example, under current regulation, how will the epidemic develop? More importantly, what is the effective policy for huge cities to fight against fast-spreading COVID-19 new variant? Responding to these questions, we constructed a system dynamic model to predict the COVID-19 trend in Shanghai and tested different policy tools to figure out effective measures for future policy decision-making.

## Materials and methods

### Data collection

Two kinds of data were used in this research. First, we tracked the policies implemented, such as contact tracing and quarantine, isolation of areas concerned, and PCR testing, of which the implementation time schedule and the scope were collected in order to set model parameters accordingly. Second, the epidemic data of COVID-19 for this wave (Mar 24^th^ to Apr 10^th^) were collected from the website of the Shanghai Municipal Health Commission, which was publicly published (19). The data included the following items: (1) daily counts of new confirmed cases, such as asymptomatic cases and symptomatic cases; (2) cumulative counts of confirmed cases; and (3) the number of people in hospital for symptomatic cases and the number of people under observation for asymmetrical cases. These data were required for model validation.

### Model structure

Susceptible-infected-recovered (SIR), and the related susceptible-infected-susceptible (SIS), susceptible-exposed-infected-susceptible-recovered (SEIR), models are typical mathematical models investigating the evolution of a disease over time. These models are based on a set of ordinary differential equations (ODEs), which presume a well-mixed population (20). The features of the epidemic determine which model to use. If the recovered populations do not become immune to the disease, the SIS model should be considered. If the epidemic has a significant incubation period, the exposed population should be added. Extensions of these classic models have been developed to incorporate human behavior, government policies, or other specific conditions (21). For example, the SVMBIR model includes an individual's decision on vaccination (V), intermediate-defense-measure (M), and both of them (B) (22). The SVEIR model has been used to study quarantine or isolation (23). The SVnIR2n model is used to study the impact of waning effect of vaccination on the spread of the disease (24).

**Figure 1:**
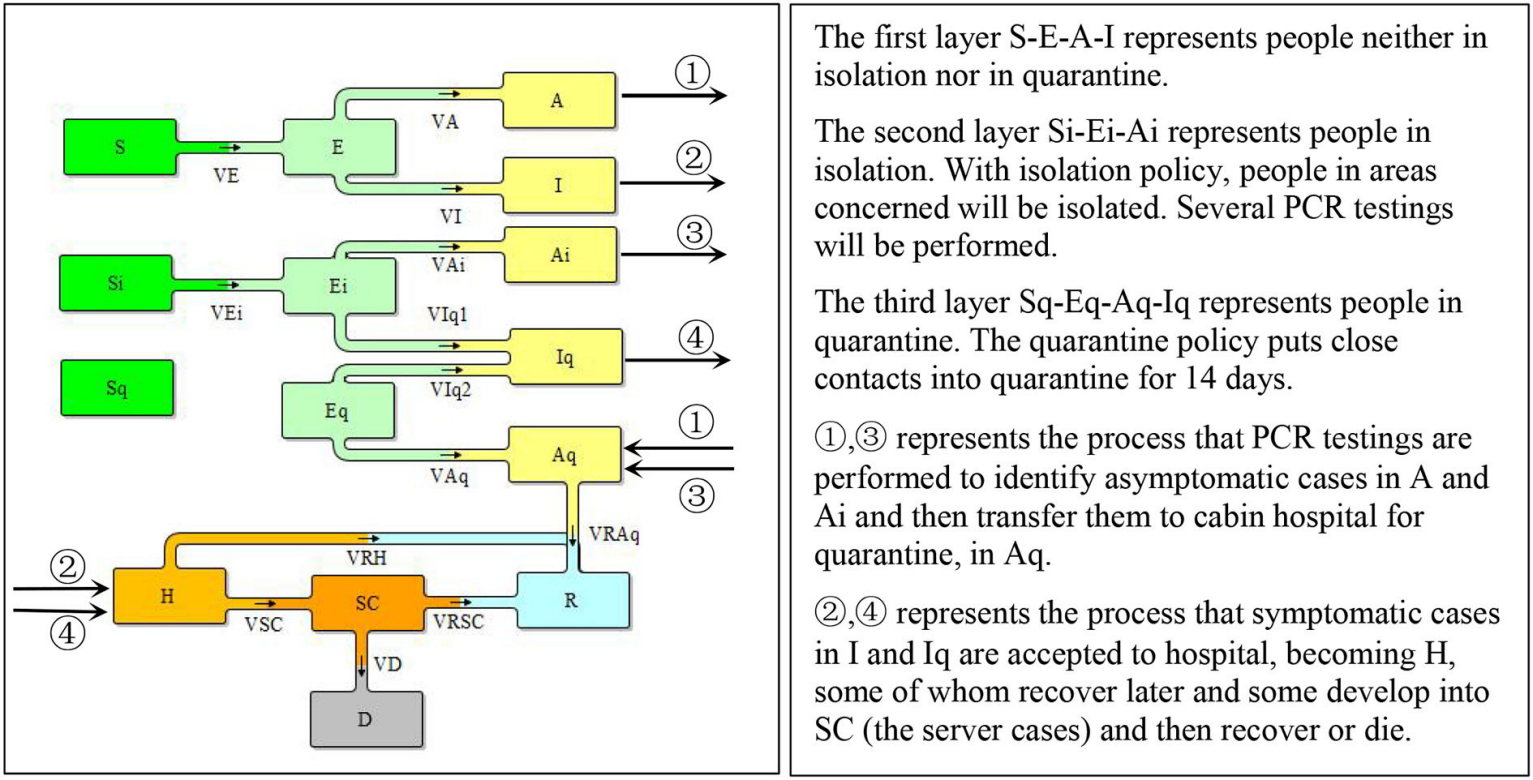
The structure of Shanghai Omicron epidemic model.

To incorporate Shanghai's policy of central quarantine of close contacts and isolation of areas concerned such as residence building or working place, we extended the SEIR model to disaggregate the total population into three layers, people who are in normal condition, people who are isolated with certain areas and people who are in central quarantine. Moreover, considering the enormous asymmetric cases caused by Omicron, in this model, we also differentiated two types of infected population *A* (asymptomatic cases) and *I* (cases with COVID-19 symptoms). Therefore, in each layer of population, there were *S* (the susceptible), *E* (the exposed), *A* (the asymptomatic cases), and *I* (the infected), as illustrated in Figure 1.

### Model equations

The Omicron spread process was represented by *V*_*E*_ and *V*_*Ei*_ where the *S* or *Si* contact with *E* and *A* or *Ei* and *Ai*. We noticed that people under isolation still contacted other persons within the isolation area, such as getting deliveries, taking part in PCR testing, and other necessary living or working activities. This made it possible for the virus to spread within the isolation area even though the contact rate was lower than that in outside the isolation areas. People under quarantine did not spread the virus anymore.


(1) $$\begin{array}{l} V_{E}=c*\beta *S*(A+I)/N*\theta *(1-\eta_{i})\\ \;+c*\beta *S*(A+I)/N*(1-\theta ) \end{array}$$

(2) $$\begin{array}{l} V_{Ei}=ci*\beta *Si*Ai/N*\theta *(1-\eta_{i})\\ \;+ci*\beta *Si*Ai/N*(1-\theta ) \end{array}$$

where c and ci represent the contact rate of the people outside isolation and under isolation, respectively; β represents the probability of transmission; N presents the total population; θ represents the vaccination rate; and η_i_ represents the effectiveness of vaccination against infection.

The *E*, will developed into asymptomatic cases, *A*, and symptomatic cases, *I*, and similarly for *Ei* and *Eq* expect that *Ei*, as already in isolation, when develop into symptomatic cases, will be immediately quarantined, which transferred into *Iq*.


(3) $$\begin{array}{r} V_{A}=\text{E*}\alpha \text{/}{\text{T}}_{\text{in}}\;V_{I}=\text{E*(1-}\alpha \text{)/}{\text{T}}_{\text{in}} \end{array}$$

(4) $$\begin{array}{r} V_{Ai}=\text{Ei*}\alpha \text{/}{\text{T}}_{\text{in}}\;V_{Iq1}=\text{Ei*(1-}\alpha \text{)/}{\text{T}}_{\text{in}} \end{array}$$

(5) $$\begin{array}{r} V_{Aq}=\text{Eq*}\alpha \text{/}{\text{T}}_{\text{in}}\;V_{Iq2}=\text{Eq*(1-}\alpha \text{)/}{\text{T}}_{\text{in}} \end{array}$$

where α represents the percentage of asymptomatic cases; therefore (1-α), was the percentage of cases with symptom, and T_in_ represents the incubation period.

The infected people without symptoms, A and Ai, could only be identified with COVID-19 testing. When confirmed to be infected, they would be transferred to cabin hospitals for quarantine and when they recover, they will be released from the cabin hospitals. V_Aq1_ and V_Aq2_ represent the flow of ① and ③, respectively, in Figure 1.


(6) $$\begin{array}{r} V_{Aq1}=A*\tau_{1}\;V_{Aq2}=Ai*\tau_{2} \end{array}$$

(7) $$\begin{array}{r} V_{RAq}=Aq1*\gamma_{Aq} \end{array}$$

where τ_1_ *and τ*_2_ represents the transfer rate of A and Ai, respectively, which are related to the testing capacity and time for other related transfer process; and γ_*Aq*_ represents recover fraction of asymptomatic cases.

The people with symptom would be received in hospital. Overtime, some would recover and some would deliver into severe cases, who would get recover or die. V_IH_ and V_IqH_ represent the flow of ② and ④, respectively, in Figure 1.


(8) $$\begin{array}{r} V_{IH}=\text{I*}\kappa \;V_{IqH}=\text{Iq*}\kappa \end{array}$$

(9) $$\begin{array}{r} V_{SC}=H*\upsilon *\theta *\left(1-\eta_{s}\right)+H*\upsilon *\left(1-\theta \right) \end{array}$$

(10) $$\begin{array}{r} V_{RH}=H1*\gamma_{H}\;V_{RSC}=SC*\gamma_{SC} \end{array}$$

(11) $$\begin{array}{r} V_{D}=SC*\phi \end{array}$$

where κ represents the hospital acceptance rate, which is related to the hospital capacity; ν represents severe case fraction and η_s_ represents the effectiveness of vaccination against severe case; γ_*H*_ represents recover fraction of hospital patients; γ_*SC*_ represents recover fraction of severe cases; ϕ represents death fraction. Details of the model equations are listed in Appendix section model structure and parameter settings are listed in Appendix section parameter setting, Tables 2, 3 in Appendix.

### Model validation

**Figure 2:**
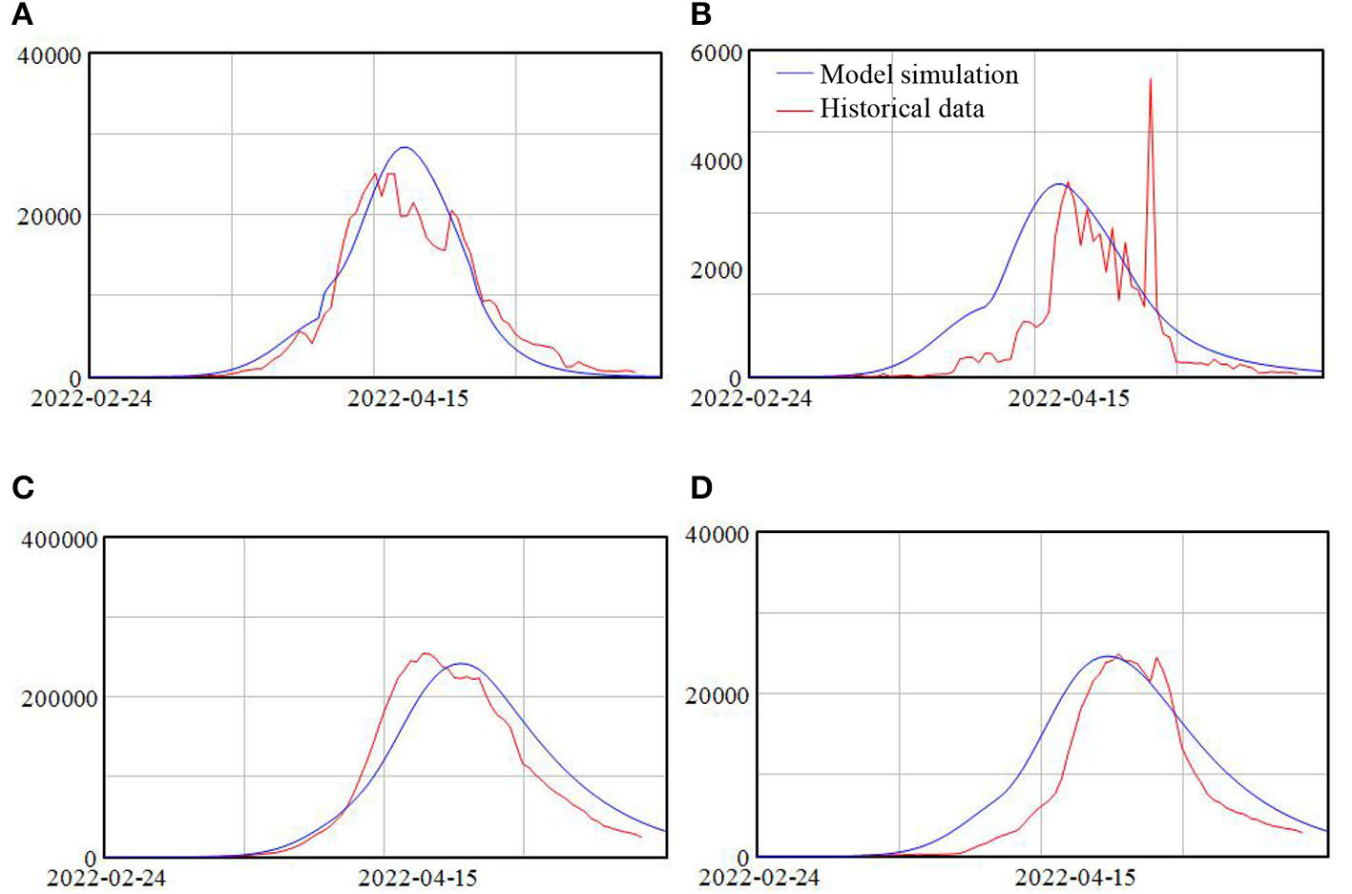
**(A–D)** Model simulation result and historical data.

The SEIR model and its extension have been widely used for the studying the spread of epidemic (25), especially for COVID-19 (26–29). This extension of the model and the parameter setting are based on the policies implemented in Shanghai, with details in Appendix. Finally, the model simulation results are compared with the epidemic data, as shown in Figure 2. Fitting the real data adds confidence to model validity.

## Results

Based on the Shanghai Omicron epidemic model, we performed simulation to investigate the effect of three policies, COVID-19 testing, isolation, and vaccination.

### Policy 1 COVID-19 testing

**Figure 3:**
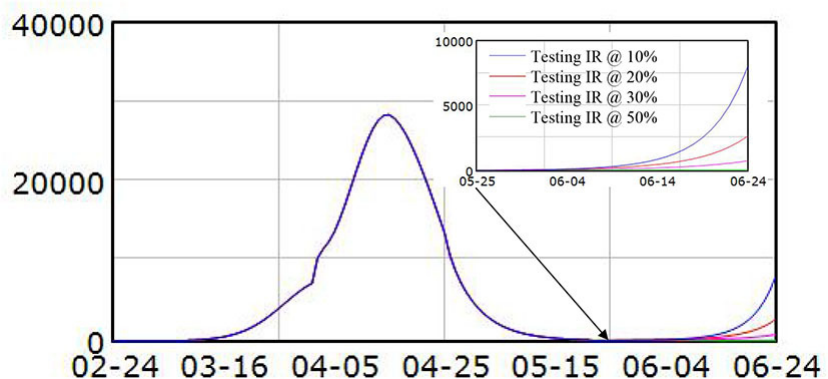
Simulation results for asymptomatic cases under different testing policies.

Four scenarios were tested with identification rate set at 10, 20, 30, and 50% after May 22nd. Results showed that if the identification rate dropped to 10%, within a month, the new infected asymptomatic cases would surge again and approach 8,000, as shown in Figure 3. With higher identification rate, the new infected asymptomatic cases would not reach that high. For the identification rate at 20 or 30%, the asymptomatic cases would increase to around 2,600 and 770, respectively. When the identification rate remained at 50%, the asymptomatic cases would be <100. Similar simulation results were identified for symptomatic cases. This simulation result illustrated that the possible way of sustainable control of the COVID-19 was high identification rate.

### Policy 2 isolation of areas concerned

**Figure 4:**
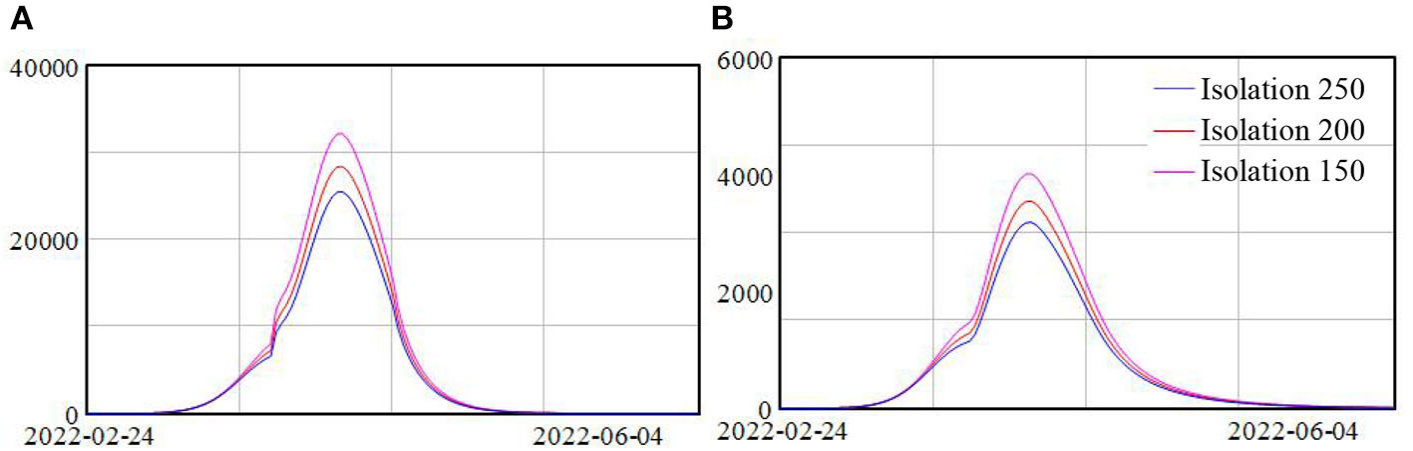
**(A,B)** Model scenarios with different isolation policy.

In this model, we used the average number of people isolated for each confirmed case or close contact as an index of the scope of the isolation policy. Three scenarios were tested, with the average number of people isolated at 250, 200, and 150, respectively. Simulation results showed that the patterns of the spread of the Omicron remained the same for three scenarios, with a slow increase at the beginning, following a fast increase, and then a decrease, as shown in Figure 4. Yet the higher the average number of people isolated, the lower the peak of the daily confirmed cases. In the scenario with the least isolation scope that is on average only 150 people isolated per each risky source, the numbers of daily confirmed asymptomatic and symptomatic cases reached more than 30 and 3.7 K, respectively. While in the scenario with the most isolation scope, the number of daily confirmed asymptomatic and symptomatic cases reduced to 25.5 and 3.2 K, representing a drop of 18 and 16%, respectively, compared with the scenario with least isolation.

### Policy 3 vaccination rate

**Figure 5:**
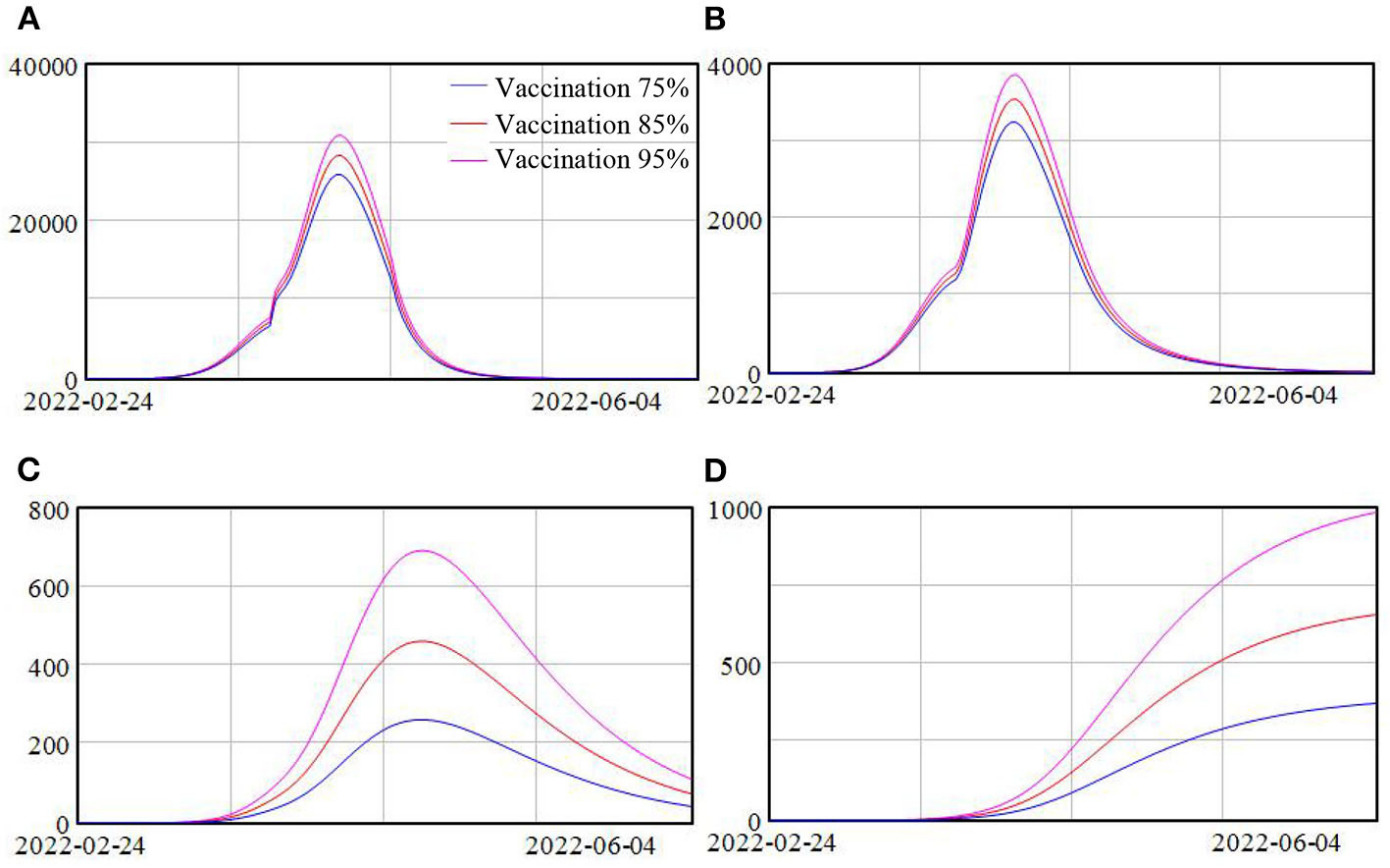
**(A–D)** Model scenarios with different vaccination rate.

Vaccination has been widely implemented in Shanghai and we investigate the impact of various vaccination rates on this wave of Omicron variant. Three scenarios were simulated with vaccination rate at 75, 85, and 95%. The simulation results are shown in Figure 5.

Due to the fact that the effectiveness of vaccination against infection for this new variant Omicron is not high, increasing the vaccination rate from 75 to 95% only slightly reduced the peak of the confirmed cases, with around 19% drop, as shown in Figures 5A,B. However, because of the high effectiveness of vaccination against severe cases, the peak of severe cases reduced significantly, from around 700 cases when the vaccination rate was 75% to around 260 cases when the vaccination rate was 95%, representing almost 170% drop. And the death population reduced from around 1,000 to 370 when the vaccination rate increased from 75 to 95%, as shown in Figure 5C.

## Discussion

Faced with the Omicron new variant, especially with BA. 2 subvariant, the most effective policy is testing. As the Omicron BA.2 is more transmissible and milder in symptoms, the former COVID-19 control essentials “early detection, early reporting, early isolation, early treatment” become harder to achieve. Fever clinics become less effective in identifying infected populations as increasing number of people do not have fever or only have a little bit fever without the need to visit clinics. The main method for identifying the infected population is now COVID-19 testing (30). In Shanghai, PCR used to be the only method for COVID-19 testing. PCR testing provides relatively accurate results but is time- and people-consuming. For a metropolis like Shanghai with 24 million residents, even with the support of medical staffs from other provinces, it took 2 days to conduct COVID-19 testing for the entire population and at least another day to get all the results. As the serial interval of Omicron might be <3 days (31), during the time period of PCR, the infected population would have already been doubled, with new exposed population remained in the population to further develop into infected population and spread the virus around. RAT has recently been applied for identifying infected people as it is fast, inexpensive, and laboratory-independent (30). However, the sensitivity of RAT to detect the COVID-19 variants has been questionable. Independent laboratory evaluations showed variable results (32–34). Caution should be taken when using RAT as a virus detection strategy. A combination of RAT with PCR test should be considered. On one hand, those who are tested positive with RAT should be re-examined using PCR. On the other hand, populations with high contagious risks should use PCR tests. In this way, early detection of the people infected by Omicron could be better achieved.

Isolation of the areas concerned has helped to reduce the peak of the daily confirmed cases, but the effectiveness of this policy is limited. Several reasons exist: (1) the isolation of the involved areas cannot fully cut the contact with the outside, for example, delivery of living necessities might bring the virus to the receiver; (2) internal movement still occurs, for instance, community workers or volunteers might get into contact with community residents when providing living support, such as maintenance or delivering services; (3) It takes one-or-two-day's time to re-confirm the COVID-19 testing result if one's test turns out to be positive, during which in the infection people might spread the virus to their family members or nearby neighbors. Besides, in the early stage, the healthcare system could not provide for enough hospital beds for the daily new confirmed cases, which might delay the transfer process as well. Our model implicated that immediate isolation of the infected people, either by transferring to a centralized quarantine location or by home isolation, can cut the infection chain more effectively.

Simulation results illustrated that increasing vaccination will not affect the new confirmed cases much, but has a significant impact on reducing the severe cases and death. The antigenic changes of Omicron reduced the sensitivity of this virus to antibody neutralization, which is responsible for the breakthrough cases (30). In this case, even though Shanghai has a high vaccination rate, the spread of the virus was still fast. In Shanghai, currently, more than 95% of the residents have completed 2 doses of vaccination and more than 10 million people have taken the booster shot (25). It is the similar case in Hong Kong, where more than 80% of the population has completed the second dose and more than one-third had taken the booster shot. The fifth wave of Omicron hit Hong Kong badly. Moreover, the death rate in Hong Kong was extraordinary high due to the low vaccination rate of the elderly population. Approximately, 95.8% of deaths were from ages above 60, a large portion of whom has not completed the second dose (35). Vaccination has proved to be effective against severe cases. It is important to improve the vaccination rate, especially for the vulnerable population group, such as the elderly.

## Conclusions

Currently, many countries have gradually ceased their restrictive policies, considering the enormous impact on the national economy and daily life. Facing the future, China also needs to look for policies that better balance COVID-19 and other economic and social issues, especially when SARS-CoV-2 new variants become less severe. The major challenge is the rapidly surging infected patients overwhelming the healthcare system. This study showed that high level of testing capacity with a combination of RAT and PCR testing to identify the asymptomatic cases. A combination of immediate home-isolation and fast transfer of confirmed cases is the key to cut the infection chain and reduced the daily infection. Moreover, to promote the vaccination in vulnerable populations could significantly reduce the severe case. These policies could be applicable to all metropolises with huge populations facing high transmissible low severity epidemic.

## Data availability statement

The original contributions presented in the study are included in the article/Supplementary material, further inquiries can be directed to the corresponding author.

## Author contributions

YQ and JH: conceptualization and writing—original draft. SC and YY: data curation. YQ, LZ, and JH: formal analysis. JH: funding acquisition and project administration. YQ and LZ: investigation and methodology. YQ and SC: software, supervision, validation, and visualization. LZ, SC, and YY: writing—review and editing. All authors contributed to the article and approved the submitted version.

## Funding

This study was supported by the Science and Technology Committee of Shanghai Municipality (grants 22692192300 and 21692190200 to JH), the Science and Technology Innovation Project of Shanghai Jiao Tong University School of Medicine-Humanities and Social Sciences (grants WK2102 to JH), and Shanghai Jiao Tong University China Hospital Development Institute 2022 Hospital Management Project (grants CHDI-2022-B-31 to JH). The funder had no role in the design, data collection, analysis, interpretation, or writing of the report.

## Conflict of interest

The authors declare that the research was conducted in the absence of any commercial or financial relationships that could be construed as a potential conflict of interest.

## Publisher's note

All claims expressed in this article are solely those of the authors and do not necessarily represent those of their affiliated organizations, or those of the publisher, the editors and the reviewers. Any product that may be evaluated in this article, or claim that may be made by its manufacturer, is not guaranteed or endorsed by the publisher.

## References

[1] Del Rio C Omer SB Malani PN. Winter of Omicron-The evolving COVID-19 pandemic. JAMA. (2022) 327:319–20. 10.1001/jama.2021.24315 34935863

[2] Burki TK. Omicron variant and booster COVID-19 vaccines. Lancet Respir Med. (2022) 10:e17. 10.1016/S2213-2600(21)00559-2 34929158 PMC8683118

[3] Andrews N Stowe J Kirsebom F Toffa S Rickeard T Gallagher E. Covid-19 vaccine effectiveness against the Omicron (B.1.1.529) variant. N Engl J Med. (2022) 386:1532–46. 10.1056/NEJMoa2119451 35249272 PMC8908811

[4] Mahase E. Covid-19: Omicron and the need for boosters. BMJ. (2021) 375:n3079. 10.1136/bmj.n3079 34906956

[5] Tian D Sun Y Xu H Ye Q. The emergence and epidemic characteristics of the highly mutated SARS-CoV-2 Omicron variant. J Med Virol. (2022) 94:2376–83. 10.1002/jmv.27643 35118687 PMC9015498

[6] NDTV. Half A Million People Killed Since Omicron, It's “Really Something”: WHO (2022). Available online at: https://www.ndtv.com/world-news/coronavirus-who-says-tragic-500-000-deaths-reported-since-covid-19-omicron-2757388 (accessed April 16, 2022).

[7] Vogel G Kupferschmidt K. Early lab studies shed light on Omicron's behavior. Science. (2021) 374:1543–4. 10.1126/science.acz9878 34941398

[8] Yu HJ Cai J Deng XW Yang J Sun KY Liu HC. Projecting the impact of the introduction of SARS-CoV-2 Omicron variant in China in the context of waning immunity after vaccination. Res Square. (2022). 10.21203/rs.3.rs-1478539/v1

[9] GOV.UK. Coronavirus (COVID-19) in the UK (2022). Available online at: https://coronavirus.data.gov.uk (accessed April 16, 2022).

[10] Nature. COVID Reinfections Surge During Omicron Onslaught. (2022). Available online at: https://www.nature.com/articles/d41586-022-00438-3 (accessed April 16, 2022). 10.1038/d41586-022-00438-3 35173320

[11] The Guardian. Parts of NHS May Be Overwhelmed by COVID Wave, Admits Boris Johnson. (2022). Available online at: https://www.theguardian.com/world/2022/jan/04/parts-of-nhs-may-be-overwhelmed-by-covid-wave-admits-boris-johnson (accessed April 16, 2022).

[12] Alderwick H. Is the NHS overwhelmed? BMJ. (2022) 376:o51. 10.1136/bmj.o51 35017162

[13] Azman AS Luquero FJ. From China: hope and lessons for COVID-19 control. Lancet Infect Dis. (2020) 20:756–7. 10.1016/S1473-3099(20)30264-4 32251637 PMC7129487

[14] WHO. Infection Prevention and Control During Health Care When Novel Coronavirus (nCoV) Infection Is Suspected. (2020). Available online at: https://www.who.int/publications/i/item/10665-331495 (accessed April 16, 2022).

[15] Salzberger B Glück T Ehrenstein B. Successful containment of COVID-19: the WHO-Report on the COVID-19 outbreak in China. Infection. (2020) 48:151–3. 10.1007/s15010-020-01409-4 32185635 PMC7095462

[16] Worldometer. COVID-19 Coronavirus Pandemic: Reported Cases and Deaths by Country or Territory. (2022). Available online at: https://www.worldometers.info/coronavirus/#countries (accessed April 16, 2022).

[17] Chen JM Chen YQ. China can prepare to end its zero-COVID policy. Nat Med. (2022) 28:1104–5. 10.1038/s41591-022-01794-3 35383312

[18] Shi Y Jiang HL Yang MX Dong LJ Chen Y Zhou YB. The precision of epidemiological investigation of COVID-19 transmission in Shanghai, China. Infect Dis Poverty. (2021) 10:58. 10.1186/s40249-021-00849-w 33947468 PMC8096468

[19] Shanghai Municipal Health Commission. COVID-19 Bulletin (in Chinese). (2020). Available online at: https://wsjkw.sh.gov.cn/yqtb/index.html (accessed April 16, 2022).

[20] Jun Tanimoto. Sociophysics Approach to Epidemics. Singapore (2021).

[21] Tanimoto J. Evolutionary Games with Sociophysics. Singapore (2018). 10.1007/978-981-13-2769-8

[22] Alam M Kuga K Tanimoto J. Three-strategy and four-strategy model of vaccination game introducing an intermediate protecting measure. Appl Math Comput. (2019) 346:408–22. 10.1016/j.amc.2018.10.015

[23] Alam M Kabir KMA Tanimoto J. Based on mathematical epidemiology and evolutionary game theory, which is more effective: quarantine or isolation policy? J Stat Mech Theory Exp. (2020) 033502. 10.1088/1742-5468/ab75ea

[24] Kabir KMA Tanimoto J. Analysis of individual strategies for artificial and natural immunity with imperfectness and durability of protection. J Theoret Biol. (2021) 509:110531. 10.1016/j.jtbi.2020.110531 33129951

[25] Halfmann PJ Kuroda M Maemura T Chiba S Armbrust T Wright R. Efficacy of vaccination and previous infection against the Omicron BA.1 variant in Syrian hamsters. Cell Rep. (2022) 28:110688. 10.1016/j.celrep.2022.110688 35421378 PMC8958134

[26] Darabi N Hosseinichimeh N. System dynamics modeling in health and medicine: a systematic literature review. Sys Dyn Rev. (2020) 36:29–73. 10.1002/sdr.1646

[27] Qian Y Xie W Zhao J Xue M Liu S Wang L. Investigating the effectiveness of re-opening policies before vaccination during a pandemic: SD modelling research based on COVID-19 in Wuhan. BMC Public Health. (2021) 21:1638. 10.1186/s12889-021-11631-w 34493226 PMC8423339

[28] Zhao J Jia J Qian Y Zhong L Wang J Cai Y. COVID-19 in Shanghai: IPC policy exploration in support of work resumption through system dynamics modeling. Risk Manag Healthc Policy. (2020) 13:1951–63. 10.2147/RMHP.S265992 33116976 PMC7550726

[29] Hou C Chen J Zhou Y Hua L Jia E. The effectiveness of quarantine of Wuhan city against the Corona virus disease 2019 (COVID-19): a well-mixed SEIR model analysis. J Med Virol. (2020) 92:841–8. 10.1002/jmv.25827 32243599

[30] Osterman A Badell I Basara E. Impaired detection of omicron by SARS-CoV-2 rapid antigen tests. Med Microbiol Immunol. (2022) 211:105–17. 10.1007/s00430-022-00730-z 35187580 PMC8858605

[31] Song JS Lee J Kim M Jeong H Kim MS Kim SG. Serial intervals and household transmission of SARS-CoV-2 Omicron variant, South Korea, 2021. Emerg Infect Dis. (2022) 28:756–9. 10.3201/eid2803.212607 35107418 PMC8888239

[32] Bekliz M Adea K Essaidi-Laziosi M Sacks JA Escadafal C Kaiser L. SARS-CoV-2 rapid diagnostic tests for emerging variants. Lancet Microbe. (2021) 2:e351. 10.1016/S2666-5247(21)00147-6 34223400 PMC8241290

[33] Schildgen V Demuth S Lüsebrink J Schildgen O. Limits and opportunities of SARS-CoV-2 antigen rapid tests: an experienced-based perspective. Pathogens. (2021) 10:38. 10.3390/pathogens10010038 33466537 PMC7824818

[34] Dinnes J Deeks JJ Berhane S Taylor M Adriano A Davenport C. Rapid, point-of-care antigen and molecular-based tests for diagnosis of SARS-CoV-2 infection. Cochrane Database Syst Rev. (2021) 3:CD013705. 10.1002/14651858.CD013705 33760236 PMC8078597

[35] Cheung PH Chan CP Jin DY. Lessons learned from the fifth wave of COVID-19 in Hong Kong in early 2022. Emerg Microbes Infect. (2022) 11:1072–8. 10.1080/22221751.2022.2060137 35348429 PMC9004509

