# Supplementary material for: Policy choices for Shanghai responding to challenges of Omicron
Source: Front Public Health. 2022 Aug 9;10:927387. doi: 10.3389/fpubh.2022.927387 (PMC9395601; doi:10.3389/fpubh.2022.927387)
Supplement: Supplementary file 1 [file Data_Sheet_1.docx]

**Supplementary appendix**

# Model Description and Definitions

In this section, we provide a detailed description of our model, including model structure, equations, and parameter setting procedures.

## Model structure

### 1.1.1 The Shanghai Omicron model

The whole model structure is presented in Figure 1 with the detailed explanation of the equations.

The total population are stratified into the following groups:

1. **S** represented the susceptible population, and **Sq, Si** reflected those in quarantine and in isolation respectively;
2. **E** represented the exposed population in the incubation period, and **Ei**, **Eq** reflected those in quarantine and in isolation respectively;
3. **A** represented the infected population without symptoms, and **Ai, Aq** reflected those in quarantine and in isolation respectively;
4. **I** represented the infected population with symptoms, and as all **I** are quarantined, **Iq** reflected those in quarantine;
5. **H** represented the population accepted in a hospital, the infected population with symptoms were required to be hospitalized; **SC** represented the population developed into severe cases.
6. **R** represented the recovered population, and **RA**, represents the recovered population from unidentified asyptomatic cases.


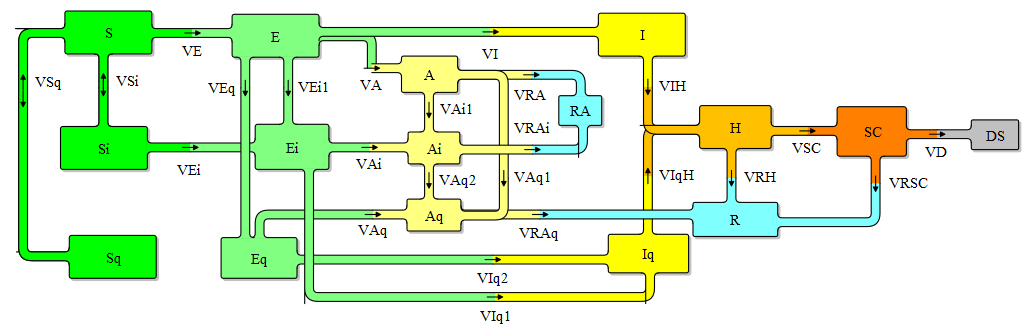


Figure 1 The structure of Shanghai Omicron epidemic model

In this model, **A and I** could transmit the disease when contacting with **S,** and **Ai** transmit the desease when contacting with **Si**. The spread of virus are illustrated by V_E_ and V_Ei_. When exposed, people will develop into symptomatic cases (V_I_, V_Iq1_ and V_Iq2_) or asymptomatic cases (V_A_, V_Ai_ and V_Aq_), and then received in hospital (V_IH_ and V_IqH_) or received in cabin hospital for observation. The hospitalized population will recover over time (V_RH_) or develop into severe cases (V_SC_), who will recover (V_RSC_) or die (V_D_). People under observation will be release when they recover (V_RAq_). If not identified early enough, over time, asymptomatic cases will recover by themselves (V_RA_ and V_RAi_).

| Discription | Model Equations |
| --- | --- |
| $S\to E$ | $V_{E}=c*\beta*S*(A+I)/N*\theta*(1-\eta_{i})+c*\beta*S*(A+I)/N*(1-\theta)$ |
| $S\to E_{i}$ | $V_{Ei}=ci*\beta*Si*Ai/N*\theta*(1-\eta_{i})+ci*\beta*Si*Ai/N*(1-\theta)$ |
| $E_{(i,q)}\to A_{(i,q)}$ | $V_{A}=\text{E*}\alpha\text{/T}_{\text{in}}$ $V_{Ai}=\text{Ei*}\alpha\text{/T}_{\text{in}}$ $V_{Aq}=\text{Eq*}\alpha\text{/T}_{\text{in}}$   |
| $E_{(i,q)}\to I_{(i,q)}$ | $V_{I}=\text{E*(1-}\alpha\text{)/T}_{\text{in}}$ $V_{Iq1}=\text{Ei*(1-}\alpha\text{)/T}_{\text{in}}$ $V_{Iq2}=\text{Eq*(1-}\alpha\text{)/T}_{\text{in}}$   |
| $I/Iq\to H$ | $V_{IH}=\text{I*}\kappa$ $V_{IqH}=\text{Iq*}\kappa$  |
| $H\to SC$ | $V_{SC}=H*\upsilon*\theta*(1-\eta_{s})+H*\upsilon*(1-\theta)$ |
| $\begin{matrix} H/SC/Aq/A \\ /Ai\to R \end{matrix}$ | $V_{RH}=H*\gamma_{H}$ $V_{RSC}=\text{SC*}\gamma_{\text{SC}}$ $V_{RAq}=Aq*\gamma_{Aq}$ $V_{RA}=A*\gamma_{A}$ $V_{RAi}=Ai*\gamma_{A}$      |
| $SC\to D$ | $V_{D}=\text{SC*}\varphi$ |

The transfer of **S** to **Sq** and **E** to **Eq** relates to the contact tracing and quarantine policy. The people that need to conduct contact tracing are those confirmed symptomatic cases and asyptomatic cases that are not in quarantine. Therefore, the contact tracing base, ConTr=V_IH_+V_Aq1_+V_Aq3_, and the contact they made is Contr*c, within which some are exposed and the others remain susceptible. For the susceptibles, after the observation period, they will be released from the quarantine and transfer back from **Sq** to **S**. Set the quarantine rate as q and the duration of observation as T_ob_, thus:

| $V_{Sq}=(ConTr*c*(1-\beta)*S/N*\theta*(1-\eta_{i})+ConTr*c*(1-\beta)*S/N*(1-\theta))*q-Sq/T_{ob}$ |
| --- |
| $V_{Eq}=(ConTr*c*\beta*S/N*\theta*(1-\eta_{i})+ConTr*c*\beta*S/N*(1-\theta)*q$ |

The transfer of **S** to **Si**, **E** to **Ei** and **A** to **Ai** relates to the isolation policy, which isolates the areas that the confirmed symptomatic cases and asyptomatic cases and their close contact have been. Therefore, isolation base Isbase= V_IH_+V_Eq_+V_Aq3_. We use the average number of people isolated, $\lambda_{a}$ for each people in isolation base as an index of the scope of the isolation policy. For the susceptibles, after the isolation period, they will be released from the quarantine and transfer back from **Si** to **S**. For exposed and asyptomatic cases, the number of people isolated is according to fraction of S has been isolated. Set the duration of isolation as T_is,_ thus:

| $V_{Si}=Isbase*\lambda_{\alpha}-Si/T_{is}$ |
| --- |
| $V_{Ei1}=V_{Si}/S*E$ |
| $V_{Ai1}=V_{Si}/S*A$ |

The transfer of A and Ai to Aq represent the testing capacity and the capacity to transfer the identified asymatic cases to centralized quarantine place. Set τ_1_, τ_2_ as the testing of people that are outside isolation or under isolation, thus:

| $V_{Aq1}=A*\tau_{1}$ $V_{Aq2}=Ai*\tau_{2}$  |
| --- |

## Parameter setting

Two types of parameters required value setting: 1) the initial value of the population groups; 2) the scenario setting variables that remained constant in one scenario and could change in different scenarios

### 1.2.1 The initial value of the population groups

The initial value of the population groups was mostly set based on the data published by the national health commission of the People’s Republic of China. Some of them were estimated based on the epidemic data. Detailed information were provided in Table 2.

Table 2 The initial value of population groups

| **Population group** | **Initial Value** | **Unit** | **Source and Explanation** |
| --- | --- | --- | --- |
| S: Susceptible population not in quarantine or isolation | 24.2378 M | person | Calculated based on the equation of total population. |
| Sq: Susceptible population in quarantine | 0 | person | No quarantine at the initial time point. |
| Si: Susceptible population in isolation | 0 | person | No isolation at the initial time point. |
| E: Exposed people not in quarantine or isolation | 3 | person | As the incubation period is three-day, one asyptomatic cases idenfied implies that there were three exposed people at least. |
| Eq: Exposed people in quarantine | 0 | person | No quarantine at the initial time point. |
| Ei: Exposed people in isolation | 0 | person | No isolation at the initial time point. |
| A: asyptomatic cases not in quarantine or isolation | 1 | person | Epidemic data released.^1^ |
| Aq: asyptomatic cases in quarantine | 3 | person | Epidemic data released.^1^ |
| Ai: asyptomatic cases in isolation | 0 | person | No isolation at the initial time point. |
| I: Infected people with sympotoms but not in quarantine | 0 | person | Epidemic data released.^1^ |
| Iq: Infected people with sympotoms in quarantine | 0 | person | No quarantine at the initial time point. |
| H: Hospitalised population | 0 | person | No hospitalised population of this wave at the initial time point. |
| SC: The severe cases | 0 | Person | No severe cases at the initial time point |
| RM: The recovered population from hospital | 0 | person | No recovered population at the initial time point. |
| RI: The recovered population without hospital treatment | 0 | person | No self-recovered at the initial time point. |
| RS: The recovered population from severe cases | 0 | person | No people recovered from severe cases at the initial time point. |
| DS: Deaths from severe cases | 0 | person | No death at the initial time point. |

### 1.2.2 Variables related to the feature of corona virus

The scenario setting variables are mostly related to the feature of the corona virus, such as transmission probability, β, the incubation period 1/ σ, the infectiousness in incubation period, θ, and the recover rate and death rate without hospital treatment γ_I_ and α_I_. New variant scenarios would change the value of one or more of these variables. The setting of base scenario were provided in Table 3.

Table 3 Variable settings for Omicron virus

| **Variables** | **Value** | **Unit** | **Source and Explanation** |
| --- | --- | --- | --- |
| N: Total population | 2.42378e+07 | person | Demographic data of the Bureau of Statistics.^2^ |
| c：contact rate | 14.8 | times/ day | The normal contact rate was 14.8.^3^ As intervention policies were implemented, the contact rate gradually dropped to the lowest value 4 when the whole city started home-isolation. |
| ci：contact rate for isolated population | 10 | times/ day | The contact rate for isolated people was lower than the normal population. But people still get contacted in the neighborhood community or workplace. The contact rate dropped to the lowest value 4 when the whole city started home-isolation. |
| q: Quarantine fraction of close contact | 0.9 | % | Shanghai has implemented the timely and precise contact tracing, which can identify most of close contact. ^4,5^ |
| β: Infectivity probability | 0.126 | 1/ times | Omicron virus doubling at 2-3 days, which means the transmission probability is around 0.126.^6,7^ |
| σ: Transition rate | 0.33 | 1/day | The reciprocal of the incubation period, which was on average 3 days for omicron.^8^ |
| κ: Hospital acceptance rate | 1 | 1/day | With adequate hospital capacity, symptomatic cases will have be received in hospital immediately. |
| $\theta$: Vaccination rate | 0.95 | % | Most of Shanghai population has taken vaccination.^9^ |
| α: Asymptomatic ratio | 0.95 | % | The major portion of the confirmed cases were asymptomatic.^10^ |
| $\lambda_{a}$: Average people isolated | 200 | person | When one person was identified to be infected, the whole resident building will be isolated. On average, resident’s buildings are 10 floors, with 5 apartments on each floor and 4 people in each household, meaning 200 people will be isolated. |
| $\eta_{i}$: Vaccination against infection | 0.1 | % | Breakthrough cases are normal for Omicron. The effectiveness of the vaccination against infection is low, at 10%.^11^ |
| $\eta_{s}$:Vaccination against severe cases | 0.9 | % | The effectiveness of vaccination against severe cases is quite high, at 90%.^11^ |
| $\tau_{1}$: Test ratio | 0 | % | This variable changes according to the testing policy. At the beginning, testing for those who were not isolated or quarantined was not performed. Later, mass testing was conducted. |
| $\tau_{2}$:Time to transfer the identified people in isolation | 2 | days | When people got positive testing result, they need to be rechecked before transfer. At least one more day is needed for arrangement of the transfer. Sometimes, it takes longer. We use the average time, which is 3 days. |
| $\nu$:Severe fraction | 0.003 | % | Omicron leads to mostly mild cases. the severe fraction is set at a low level.^11^ |
| γ_H_: Time to recover for symptomatic cases | 7 | day | Individuals will on average recover within 7 days. ^12^ |
| $\gamma_{A}$: Days for recover for asymptomatic cases | 5 | days | Normally, it will take 5 days for people to recover. ^13^ |
| $\gamma_{Aq}$:Observation period for asymptomatic cases | 25 | days | It was long at the beginning, but with was gradually reduced to around 14. |
| $\gamma_{SC}$:Recover fraction of Severe Cases | 0.05 | % | It took much longer for severe cases to recover, normally require average of 42 days. ^14^ |
| $T_{ob}$:Observation period for S | 14 | days | It is the quarantine policy that 14 days are required for the susceptible population to complete quarantine. ^8^ |
| $T_{in}$:Incubation period | 2 | days | The incubation period for omicron is 4.4 days.^15^ |
| $T_{is}$:Time for isolation | 7 | days | Different situation had different isolation policy, ranging from 2 days, 7 days and 14 days. On average, people were isolated for 7 days.^16^ |
| $\varphi$:Death fraction | 0.001 | % | The death fraction is relative low for Omicron. ^17^ |

# Intervention policies implemented in Shanghai

2022.4.11

Shanghai differentiated three types of zones, the isolation zone, the control zone and the precaution zone with different prevention and control.

25,173 asymptomatic cases and 914 symptomatic cases were reported.

2022.4.10

2022.4.8

2022.4.2

2022.3.13

2022.3.28

2022.3.10

2022.4.9

2022.4.6

2022.4.4

2022.4.1

2022.3.15

2022.2.24

One asymptomatic case and one symptomatic case were reported.

2022.3.28

The east areas of Huangpu Riverr implemented home-isolation while nucleic acid testing was performed.^18^

City-wide testing was carried out using either antigen self-tests or nucleic acid test.

22,609 asymptomatic cases and 1,015 symptomatic cases were reported.

A new makeshift hospital opens in Chongming to accommodate asymptomatic cases.

A city-wide COVID-19 antigen self-tests was performed.

7,788 asymptomatic cases and 38 symptomatic cases were reported.

The west areas of Huangpu River implemented home-isolation while nucleic acide testing was performed.^18^

4,381 asymptomatic cases and 96 symptomatic cases were reported.

Colleges and universities adopted the closed management mode.

2022.3.1

128 asymptomatic cases and 41 symptomatic cases were reported.

A negative nucleic acid test report was required to be into or out of Shanghai.

2022.3.12

All primary and secondary schools adjusted to online learning.

2022.3.11

64 asymptomatic cases and 11 symptomatic cases were reported.

One asymptomatic case was reported.

Figure 2 Shanghai Policy

# Incorporating the stochastic effect in the simulation

## Contact rate

We used the average contact rate in the model to illustrate the normal situation. However, different person has different contact behavior. To present the stochastic effect, we used a normal distribution in modeling the contact rate.


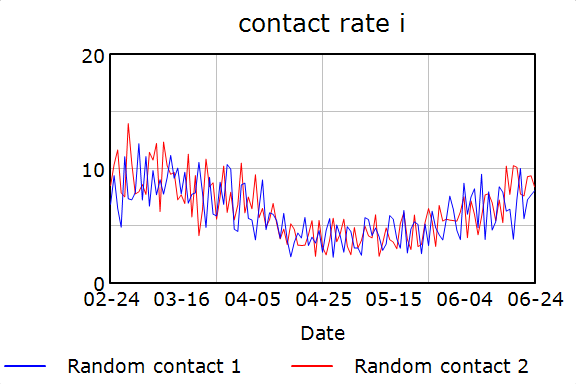

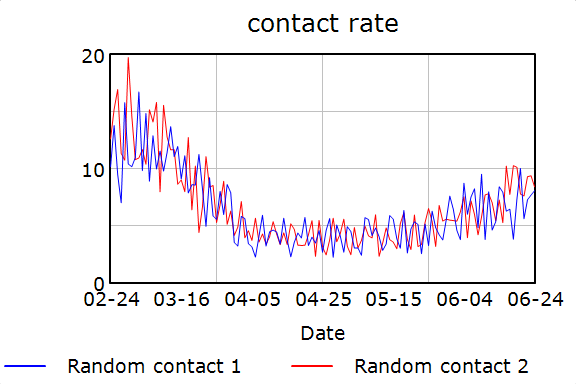


1. Contact rate outside isolation (b) Contact rate under isolation


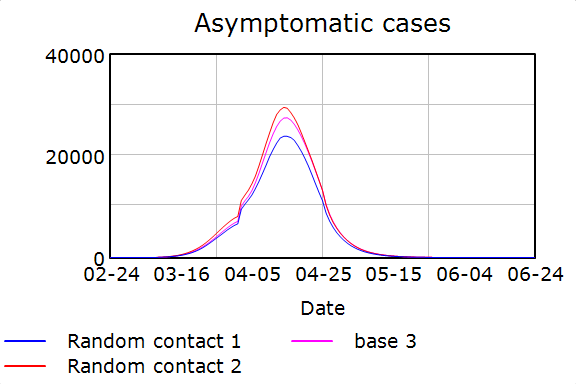


1. New confirmed cases--Asymptomatic


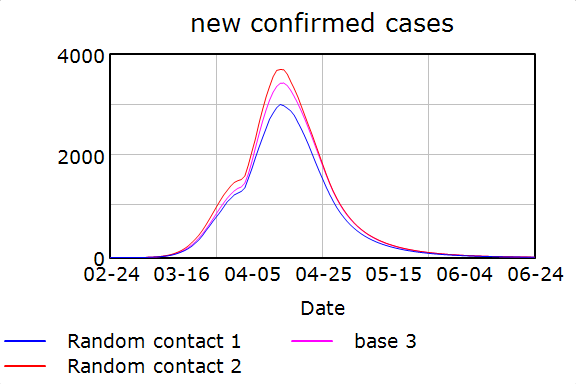


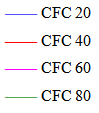


Random Factor 1

Random Factor 2

Base (without random factor)

1. New confirmed cases – Symptomatic

Simulation results show that with random factors, the new confirmed cases, both for asymptomatic cases and symptomatic cases, have not changed significant. The model simulation pattern remains the same, expect that the peak of increases or decreases slightly.

## Incubation period

Even though study showed that the average incubation period was 3 days for Omicron8, it is also widely observed that incubation period varies for different person.


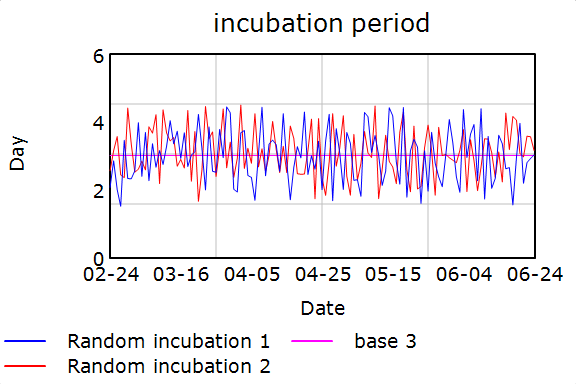


1. Incubation period


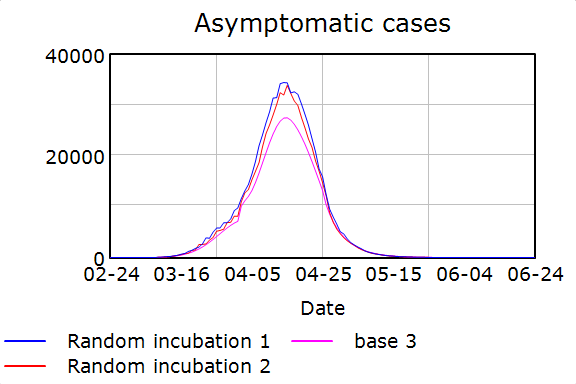


1. New confirmed cases—Asymptomatic


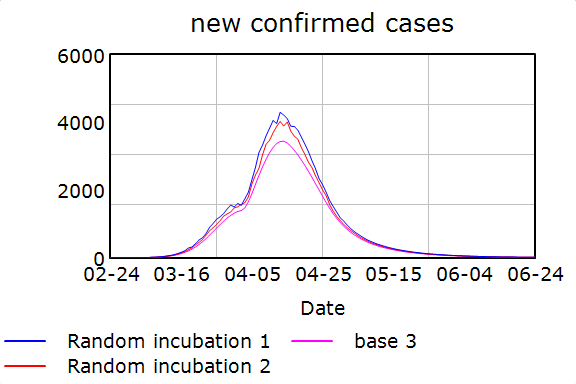


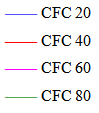


Random Factor 1

Random Factor 2

Base (without random factor)

1. New confirmed cases—symptomatic

Simulation results show that with random factors, the new confirmed cases, both for asymptomatic cases and symptomatic cases, have not changed significant. The model simulation pattern remains the same, expect that the peak increases or decreases slightly.

# Reference

1. Liu, M., 2022. On February 24 (0-24:00), Shanghai reported no new local confirmed COVID-19 cases, including 59 imported cases and 1 asymptomatic local COVID-19 case. [online] Wechat public platform. Available at: https://mp.weixin.qq.com/s/g8xeJQOYrw-FZy8Vsx7P6A [Accessed 25 February 2022].

2. Tjj.sh.gov.cn. 2022. Statistical Bulletin of National Economic and Social Development of Shanghai in 2021 statistical Bulletin of Shanghai Municipal Bureau of Statistics. [online] Available at: <http://tjj.sh.gov.cn/tjgb/20220314/e0dcefec098c47a8b345c996081b5c94.html> [Accessed 15 March 2022].

3. Tang B, Wang X, Li Q, et al. Estimation of the Transmission Risk of the 2019-nCoV and Its Implication for Public Health Interventions. J Clin Med. 2020;9(2):462. doi:10.3390/jcm902046213

4. Shanghai Municipal Health Commission. COVID-19 Bulletin (in Chinese); 2020. Available from: http://wsjkw.sh.gov.cn/yqtb/index. html. Accessed August 3, 2020.

5. Azman AS, Luquero FJ. From China: hope and lessons for COVID-19 control. Lancet Infect Dis. (2020) 20(7):756-757. doi: 10.1016/S1473-3099(20)30264-4.

6. Sharp, J. 2022. COVID-19: Omicron cases 'doubling every 2-3 days' and expected to be dominant strain within a month [online] Available at: https://news.sky.com/story/covid-19-omicron-cases-doubling-every-2-3-days-and-expected-to-be-dominant-strain-with-a-month-12490171 [Accessed 25 March 2022].

7. Du, Zhanwei & Hong, Huaping & Wang, Shuqi & Ma, Lijia & Liu, Caifen & Bai, Yuan & Adam, Dillon & Tian, Linwei & Wang, Lin & Lau, Eric & Cowling, Benjamin. (2022). Reproduction Number of the Omicron Variant Triples That of the Delta Variant. Viruses. 14. 821. 10.3390/v14040821.

8.Shanghai Municipal Health Commission. Returnees stay home for 14 days (in Chinese); 2020. Available from: http://wsjkw.sh.gov.cn/xwfb/20200204/ db01b711b12040c4bbba663289ea00f7.html. Accessed March 10, 2020.

9. Prem K, Liu Y, Russell TW, Kucharski AJ, Hellewell J. The effect of control strategies to reduce social mixing on outcomes of the COVID-19 epidemic in Wuhan, China: a modelling study. *Lancet Public Health*. 2020; **5**: E261-70.

10. Murray, C., 2022. COVID-19 will continue but the end of the pandemic is near. The Lancet, 399(10323), pp.417-419.

11. Mefsin Y, Chen D, Bond HS, et al. Epidemiology of infections with SARS-CoV-2 Omicron BA.2 variant in Hong Kong, January-March 2022. medRxiv; 2022. DOI: 10.1101/2022.04.07.22273595.

12. Xinhuanet.com. 2022. The average length of stay in Shanghai makeshift hospitals is gradually being reduced to about a week. [online] Available at: <http://www.xinhuanet.com/2022-04/13/c_1128556083.htm> [Accessed 13 April 2022].

13. Brazil, H., 2022. [online] Available at: https://www.fontoura.com/english/2022/01/07/ [Accessed 7 January 2022].

14. Health.onlineium.com. 2022. Omicron: Everything You Need To Know About Symptoms, Isolation And Recovery. [online] Available at: https://health.onlineium.com/wellness/omicron-everything-you-need-to-know-about-symptoms-isolation-and-recovery [Accessed 23 May 2022].

15.Miller, K., 2022. Omicron's Incubation Period Is Short: Here's Why That Matters. [online] Prevention. Available at: https://www.prevention.com/health/a38868608/omicron-incubation-period/ [Accessed 24 January 2022].

16. Sh.bendibao.com. 2022. What does Shanghai's 7+7 quarantine policy mean. [online] Available at: http://sh.bendibao.com/news/2022321/249960.shtm [Accessed 24 March 2022].

17. Kate, W., 2022. If Omicron is less severe, why are COVID-19 deaths rising?. [online] World Economic Forum. Available at: https://www.weforum.org/agenda/2022/01/omicron-covid19-death-toll-severity/ [Accessed 31 January 2022].

18. Kelly, N., 2022. Shanghai Enters China’s Biggest Citywide COVID-19 Lockdown. [online] That's Online. Available at: https://www.thatsmags.com/shanghai/post/ [Accessed 28 March 2022].
